# ‘I Think the First Priority is Physically Safe First, Before You Can Actually Get Psychologically Safe’: Staff Perspectives on Psychological Safety in Inpatient Mental Health Settings

**DOI:** 10.1111/jpm.13101

**Published:** 2024-09-16

**Authors:** Katharina Sophie Vogt, John Baker, Matthew Morys‐Edge, Sarah Kendal, Emily Mizen, Judith Johnson

**Affiliations:** ^1^ Temple Bank House, Bradford Institute for Health Research Bradford Royal Infirmary Bradford UK; ^2^ Department of Psychology University of Leeds Leeds UK; ^3^ School of Healthcare University of Leeds Leeds UK; ^4^ Norwich Medical School University of East Anglia Norwich UK; ^5^ School of Public Health and Community Medicine University of New South Wales Sydney Australia

**Keywords:** qualitative methodology, quality of care, safety and security, staff perceptions, staffing/resources

## Abstract

**Introduction:**

While the concept of psychological safety has been gaining momentum, research concerning psychological safety in inpatient mental health wards is lacking.

**Aim:**

To investigate how psychological safety is conceptualised by healthcare staff in inpatient mental health units, and what barriers and facilitators exist.

**Method:**

Reflexive Thematic Analysis was used to analyse 12 interviews.

**Results:**

Participants conceptualised psychological safety as feeling safe from physical harm, being able to develop meaningful relationships and feeling valued at work. Participants often did not feel physically safe at work, which led them to feel psychologically unsafe. Barriers to psychological safety were reliance on agency workers, punitive management approaches and the inherent risk in working with mental health inpatients. Facilitators included appropriate staffing ratios and skill mix, being able to form meaningful relationships and having access to support.

**Discussion:**

The emphasis on the physical safety element within psychological safety means that existing definitions of psychological safety require extension for the mental healthcare context. However, large‐scale research is needed to further understand experiences of psychological safety in this group.

**Implications for Practice:**

A better understanding of the dimensions of psychological safety in inpatient mental health settings could support the development of tools to investigate psychological safety interventions. Organisations could support psychological safety through regular staff supervision and improved staffing ratios and skill mix.

**Summary**

**What is Known on the Subject?**

- The concept of psychological safety has gained momentum, with research showing its importance in generating successful outcomes and preventing patient safety incidents/errors.
- Psychological safety has been defined as the belief that it is safe to take interpersonal risks, without a fear of negative consequences.
- However, to date, it had not been investigated in the healthcare staff working in inpatient mental healthcare.

**What the Paper Adds to Existing Knowledge?**

- This is the first study to investigate psychological safety in inpatient mental healthcare staff.
- Participants in the interviews conceptualised psychological safety as a feeling of being safe from physical harm, being able to developing meaningful relationships with service users and staff and feeling valued at work. For the participants, feeling physically safe was an especially important prerequisite for feeling psychologically safe.
- The study results show that staff on inpatient mental health wards feel neither physically nor psychologically safe, and this stops them from delivering excellent patient care.

**What are the Implications for Practice?**

- Organisations must listen to their staff and invest in ways to make these work environments more physically and psychologically safe.
- More research must be undertaken to increase understanding of how to support psychological safety of inpatient mental healthcare staff.

## 1 Introduction

On acute mental health wards, mental health problems are assessed, acute illnesses treated, a safe environment is provided, and patients are supported in their rehabilitation (Bowers et al. 2009). Reasons for admission include crisis stabilisation, aggression, assessment for, or presence of, distress, containment, protection of harm and treatment (Bowers et al. 2009; Nathan et al. 2021). Healthcare staff working in these settings, especially nurses and support workers, thus face a unique work environment of supporting individuals in crisis who often cannot keep themselves (or others) safe, while being at high‐risk of exposure to, and experience of, violence (Weltens et al. 2021). This environment has long been associated with negative outcomes for staff, including poor wellbeing, lower health‐related quality of life and compassion fatigue (Johnson et al. 2018), which have, in turn, been associated with poorer quality of care and greater risk of patient safety concerns (Johnson et al. 2018; Keers et al. 2018). Patient safety relates to the prevention and reduction of risk, errors and harm that occur while an individual is receiving healthcare (World Health Organization 2019).

While there are some shared risks to patient safety across mental and physical healthcare (e.g., misdiagnoses, medication errors), mental healthcare has unique patient safety risks (e.g., violence, aggression, self‐harm) that relate to some presentations of distress and to the ways in which this is managed (e.g., restraint, involuntary medication) (Chieze 2019; Doedens et al. 2020; Donaldson et al. 2021; Thibaut et al. 2019).

Over the past two decades, the concept of psychological safety has gained momentum: healthcare organisations increasingly recognise its importance in generating successful outcomes and preventing patient safety incidents and errors (Grailey et al. 2021; Hunt et al. 2021; Swendiman, Edmondson, and Mahmoud 2019). Traditionally, psychological safety has been defined as the belief that it is safe to take interpersonal risks, without fear of negative consequences (Edmondson 1999). This essentially conceptualises psychological safety as the feeling that employees can raise concerns (i.e., take risks), such as speaking up about patient safety concerns, naming bad practice or making suggestions for improvements, without fear of negative repercussions or a threat to their position (Okuyama, Wagner, and Bijnen 2014; Richard, Pfeiffer, and Schwappach 2021). As a multi‐level concept, psychological safety can be conceptualised at the individual, team or organisational level (Edmondson 1999; Hunt et al. 2021; Newman, Donohue, and Eva 2017).

Given higher rates of burnout and poorer staff wellbeing in mental healthcare professionals compared with other healthcare professional groups (Johnson et al. 2018), it is possible that poor psychological safety could be a pertinent issue for mental healthcare staff. Furthermore, variations in patient safety issues encountered in mental healthcare settings, together with differences in working conditions compared with other settings, suggest that variation is also possible in (1) the way psychological safety is conceptualised and (2) the factors that act as facilitators and barriers to its presence.

Furthermore, healthcare professionals often report poor experiences of physical safety in mental health inpatient units (Cranage and Foster 2022; Kelly et al. 2016; Renwick et al. 2019; Williams, Knight, and Sarfraz 2023). Staff working in acute inpatient mental health units have a higher risk of being at high‐risk for exposure to, and experience of, violence (Weltens et al. 2021). In addition, chronic exposure to (perceived) unsafe physical environments can lead to negative long‐term consequences for staff, in terms of both physical and psychological health (Kelly et al. 2016). It is possible that experiences of (a lack of) physical safety might be related to psychological safety, but as no exploration has been conducted, there is no evidence to substantiate this.

There is a dearth of research investigating psychological safety in mental healthcare staff, and no study has assessed how psychological safety is conceptualised by healthcare staff working in acute mental health units. There is also a lack of research investigating what staff consider to be key facilitators or barriers for establishing psychological safety within mental healthcare teams.

The present study aimed to address this by conducting a qualitative exploration into (1) the conceptualisation of psychological safety by acute mental healthcare staff and (2) the facilitators and barriers to its presence in inpatient mental healthcare units.

## 2 Methods

### 2.1 Design

A qualitative interview study was conducted. Online, semi‐structured interviews were deemed to be the most suitable data collection method because of their flexibility. Questions centred around (1) conceptualisations of psychological safety, (2) how different aspects of the ward environment can affect psychological safety, and (3) how to improve psychological safety in inpatient mental health wards (Appendix 1).

### 2.2 Ethical Approval

Ethical approval was granted by the University of Leeds.

### 2.3 Recruitment

A volunteer sample of 12 participants was recruited via social media (Table 1). None dropped out of the study after agreeing to take part. Participants had to be healthcare professionals who were currently working, or had previously worked, in adult inpatient mental health wards in the UK. No restrictions were made regarding healthcare role, length of time in post, or experience. Participants had no prior relationship with the interviewer. They were provided with the researcher's job title, and it was explained that the researcher was part of a wider study into psychological safety in mental health settings.

**TABLE 1:** Participant overview.

Pseudonym	Age	Occupation	Interview length (minutes) mean time = 33 min
Louisa	31	Mental health support worker (adult‐inpatient)	28
Doris	27	Mental health support worker (adult‐inpatient) [UK], Mental health nurse trained [not yet UK registered]	31
Hannah	31	Mental health nurse	31
Daisy	24	Assistant practitioner (adult‐inpatient)	28
Eleanor	26	Trainee clinical psychologist, previously healthcare assistant (adult‐inpatient)	50
Patsy	26	Assistant psychologist, previously Healthcare Assistant (adult‐inpatient)	77
Marie	27	Senior psychological wellbeing practitioner, previously healthcare assistant and assistant psychologist	25
Sarah	24	Assistant psychologist, previously healthcare assistant (adult‐inpatient)	37
Lilly	24	Assistant psychologist, research assistant	36
Lucy	27	Trainee clinical psychologist, previously occupational therapist	19
Tatjana	Missing	Junior doctor (psychiatry trainee)	12
Jill	27	Mental health nurse (with lived experience)	23

### 2.4 Procedure

Study advertisements were shared on social media. Interested participants were asked to email the Senior Research Fellow for more information. They were then sent an information sheet and given the opportunity to ask questions. Once they agreed to participate, they were sent an online consent form and a choice of dates/times for interview. Participants were asked to be in a confidential space, where they could freely talk about their experiences with no one else present. Participants selected their interview slot. KSV conducted the interviews via Zoom. Following the interview, participants were emailed a debrief sheet (containing contact numbers for support services) and a £30 shopping voucher. Interviews were transcribed via Zoom, but edited by the researchers for accuracy. The qualitative analysis program Atlast.ti was used to aid analysis (ATLAS.ti Scientific Software Development GmbH 2022); all researchers had access to the data. Interviews took place between October and November 2022 and were analysed between January and April 2023.

### 2.5 Research Team

The lead researcher (KSV) was a female, Chartered Psychologist and Post‐Doctoral Senior Research Fellow, with extensive training in qualitative research methods (MSc, PhD). The rest of the research team comprised a Clinical Psychologist (JJ), a Professor in Mental Health Nursing (JB), two PhD students in safety in mental health (BG, HS) a mental health nurse (SK), an Undergraduate Psychology student (MME) and an Assistant Psychologist (EM). Multiple people within the research team had lived experience of being patients in inpatient mental health settings. To protect researcher confidentiality, no initials are shown for those with lived experience.

### 2.6 Epistemological Framework

This study was developed from a constructionist epistemology. It included an experiential orientation to data with inductive analysis of the interviews. In combination, these approaches are consistent with the idea that people create meaning by interpreting what is happening around them.

Thus, in the current context, it is acknowledged that the participants were constructing a reality from their lived experience, which is interpreted by the researchers through their own lenses of lived experience (Byrne 2022). As the topic under investigation was likely to be personal to each interviewee, with everyone experiencing their own realities of ‘psychological safety’, an essentialist approach was unsuitable. Although a critical orientation to data is permissible within Reflexive Thematic Analysis (see description of analysis), it too was deemed inappropriate for the current study, since the study aim was to investigate the experience of social reality, consistent with an experiential framework. Both latent and semantic coding were used in the analysis; this is generally viewed as deepening the level of analysis (Braun and Clarke 2006; Byrne 2022).

### 2.7 Analysis

The team identified Reflexive Thematic Analysis (RTA) as the most suitable analysis method, appropriate for capturing participants' varied definitions and experiences of psychological safety in inpatient mental health (Braun and Clarke 2019, 2021). RTA follows six steps—Table 2 summarises how the steps of RTA were followed by the researchers. Discussions about data saturation were held between KSV and JJ after analysis of the 12 interviews; both researchers felt that data saturation was reached at this point. All authors contributed to the final review of themes. Where a finding was supported by two quotations evidencing different aspects of the finding, the quotations have been reported sequentially following the findings statement.

**TABLE 2:** Analysis process of reflexive thematic analysis (RTA).

Step of RTA	Description	How followed
Familiarisation	‘Getting to know’ the data.	KSV conducted the interviews. MME and KSV transcribed the interviews, then both read the transcripts multiple times to ensure they gained further familiarity with the interview transcripts
Generating initial codes	Capturing what is analytically interesting about the data by assigning codes to sections of data.	After reading the transcripts multiple times, KSV and MME coded two interviews by hand, comparing codes given, to ensure consistency
		Following this, all transcripts were uploaded to Atlas.ti.nd, and KSV and MME completed the first round of coding
Generating themes	Gathering codes together into meaningful units.	KSV and MME generated the first themes, in collaboration with JJ
Reviewing themes	Refining the themes.	The whole research team reviewed the themes, presented by KSV and MME
Defining & naming themes	Capturing the meaning of a theme in a few words.	KSV defined and named the themes, supported by MME and JJ
Creating the report	Writing up; relating analysis to the research question and wider literature.	KSV produced the report, supported by the whole research team

### 2.8 Reflexivity

The authors acknowledge the importance of reflexivity in the context of RTA and how our past experience may have impacted the analysis of interviews. The extent and impact of this were discussed in research team meetings. Experiences that may have impacted the analysis of the results within the research team include having previously worked in inpatient mental health wards, conducting experience in the field, or having lived experience of being an inpatient on acute mental health wards.

The COREQ (Consolidated criteria for REporting Qualitative Research Checklist) was used to guide the reporting (Appendix 1).

## 3 Results

**FIGURE 1:**
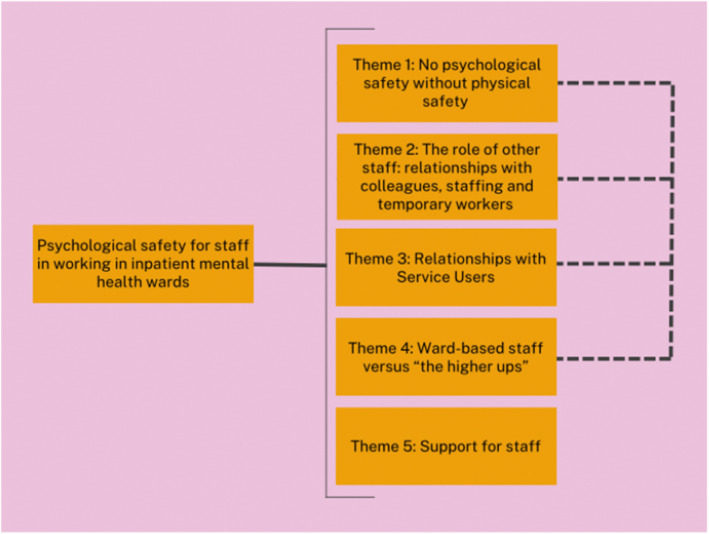
Graphic representation of the developed themes.

Five themes were developed: (1) No psychological safety without physical safety; (2) The role of staff: staffing, relationships, and skill‐mix; (3) Relationships with service users; (4) Ward‐based staff versus ‘the higher ups’; and (5) Psychological support integral to psychological safety. While these themes are distinct, the ‘physical safety’ element of Theme 1 links with Themes 2–4 (highlighted in Figure 1).

### 3.1 Theme

#### 3.1.1 No Psychological Safety Without Physical Safety

When participants were asked about things that made them feel psychologically safe, they often discussed factors which supported their physical safety in the first instance, indicating this was a prerequisite for psychological safety. Thus, the relationship between psychological safety was described as both inter‐linked, conditional and hierarchical.

priority is physically safe first, before you can actually get psychologically safe—Doris.

Participants reported often feeling physically and psychologically unsafe and acknowledged that managing service users' distress and physical risk (to the service users themselves, to others) was ‘part and parcel of the job’ (Marie). Staff spoke in detail about the trauma they had experienced or witnessed as part of a response team while working on the wards.

I don't think you feel psychologically safe […] because the patient can be really distressed, really agitated, and physically aggressive.—Daisy.

When a restraint happens… […] it very much felt like‐ it was just risky for everyone… reminds you how quickly those things can happen.—Lilly.

Participants expressed that working on wards left them feeling hyper‐aware of harms that service users can inflict on themselves via self‐injury or to staff and other service users, and that this awareness was a barrier to feeling physically and psychologically safe. One explained that this knowledge, and the anticipation that an incident can arise at any time, left her ‘feeling quite fearful and insecure at times’ because she and her team ‘were just constantly on edge, just waiting for the next thing to happen’ [Marie]. This sentiment was shared by others.

this risk of physical harm, the risk of violence, but also being shouted at is quite traumatic… not being able to get away from it, because it's your workplace or because you physically can't get away from it, and being literally stuck, and trapped, and there's no escape.—Tatjana.

Participants acknowledged that working on wards can be traumatic, and that they were impacted by past traumatic experiences. They also acknowledged certain behaviours/actions by service users may trigger memories of trauma, which might lead staff to misjudge a situation or risk and lead, for example, to a restraint rather than a verbal de‐escalation. Thus, a staff member's unresolved trauma could affect their psychological safety, which could in turn influence decision‐making that negatively impacted care.

had I lashed out or reacted from being traumatized, that's dangerous ‐ last time I almost died because of this, so I'm going to pull my alarm and we're going to restrain.—Patsy.

Participants drew links between psychological safety and physical safety, and identified physical safety as a key precursor to psychological safety. This finding is also evident across the other themes.

### 3.2 Theme 2: The Role of Other Staff: Relationships With Colleagues, Staffing and Temporary Workers

This leads directly on from Theme 1 and contains narratives around the role of other staff, including relationships with colleagues, staffing ratios and skill‐mix, as well as temporary workers.

#### 3.2.1 Relationships With Colleagues

Feelings of psychological safety were facilitated by working with colleagues with whom participants had established positive relationships. It helped further when these colleagues were experienced, permanent members of staff, whom they knew they could rely on to come to their aid. It also helped when these colleagues were responsive to the needs of their team members, communicated well and were confident in managing difficult situations. Therefore, psychological safety for participants was more about having a good, dependable team than about whether they felt safe to take risks or make suggestions for improvement.

… [patient] grabbed my arms, and another team member… recognize it so she just run to us. […]. And this is really not only physically feel safe, but also psychological safe, is that I don't need to like struggle with the patient.—Doris.

When you have a good supportive team around you, no matter the acuity ‐you feel supported, and you feel psychologically safe.—Daisy.

The link between physical and psychological safety was also evident in this theme. For example, Louisa described an instance where she worked with team members whom she did not trust to respond when she pulled her alarm in an emergency; this made her feel unsafe because she was unsure whether someone would come to her aid, and because she felt anxious about what could potentially happen. She then described working with a ‘better’ team, saying that ‘I know that if something happened and I pulled the alarm, they are coming in two seconds’ (Louisa).

#### 3.2.2 Staffing

Participants described feeling physically safer when there were enough staff on the wards, on the basis that patient care and risk levels could be managed more appropriately/safely. However, staffing alone was not enough to facilitate psychologically safety.

because of a lack of manpower‐ one time, there was just like one staff […] with around six to seven patients. […] When one of them, like really aggressive and agitated ‐ I tried to… verbally de‐escalate, but to limited effect. So, I feel both physically and psychologically unsafe.—Doris.

Not knowing where your support's going to come from, you know, if you're low on staff […] it's that fear that you're not going to manage and something terrible is gonna happen—Marie.

In addition, insufficient staff could mean that service users were not escorted to the smoking area or on leave, potentially leading to frustration and possible verbal and physical aggression, thereby reducing workers' sense of physical safety and acting as a barrier to psychological safety. Not having enough staff could also mean that personalised care did not take place, straining relationships between staff and service users.

… we didn't really have the time to make patients feel safe… we didn't even have a minute spare.—Patsy.

#### 3.2.3 Temporary Staff

There was much discussion about the role of permanent versus temporary (‘agency’) staff across the interviews. Many mental health units in the UK and elsewhere require the use of temporary workers to ensure adequate staffing levels (Tamburello, Borneo, and Holden 2023). Participants did not think temporary healthcare support workers and nurses made wards safer or facilitated psychological safety, even if it improved staffing ratios.

Participants suggested three possible explanations. First, temporary staff are usually independent of the hospital, and wards cannot control which workers they will have and when. As such, there is a lack of opportunity for agency workers to form bonds with permanent ward staff (and vice versa), where trust and mutual understanding can develop over time. Second, temporary workers were often perceived as young and inexperienced, ‘quite young and new to the field’ [Patsy], without the same level of training ‘as other staff’ [Eleanor], making participants worry whether these workers were appropriately qualified to manage situations of distress, risk, or incident, or whether their reaction may even escalate the situation. Third, having temporary staff on wards meant that permanent staff had to undertake jobs that temporary staff were unable to do, such as completing documentation. This meant they were occupied with administrative or clerical tasks, instead of being with the service users. The importance of developing good relationships with service users for the psychological safety of staff is further explored in Theme 3.

permanent staff […] have to attend to all the paperwork ‐ they lose that connection with service users, and it's just a perfect storm really. I think having that good regular team can help everyone feel safer in that environment.—Patsy.

### 3.3 Theme 3: Relationships With Service Users

Getting to know service users, their (trauma) history, establishing whether they present a risk of harm to themselves or others, and finding out ‘what's helpful and what's not’ (Eleanor) to service users was viewed as essential for facilitating a sense of psychological safety. Being able to form good relationships with service users enabled them to judge risk, also making participants feel more physically safe.

I knew that he was sort of all bark… he was saying things, but I was physically safe.—Sarah.

Participants reported that getting to know service users enabled them to provide person‐centred care, and work therapeutically with service users, which facilitated their feelings of psychological safety. However, there was an acknowledgement that, often, meaningful interaction happens off the ward, in counselling or therapy sessions, rather than on the ward during daily observations. For ward‐based staff, this might be a barrier for establishing feelings of psychological safety.

… the best bit of the [off‐ward therapy] group, is that level of connection. And that makes me feel safe, and I think it makes other people feel psychologically safe as well.—Sarah.

Within the constraints of the ward environment, there were many barriers to establishing these good, supportive relationships with service users, including poor staffing and administrative duties.

### 3.4 Theme

#### 3.4.1 Ward‐Based Staff Versus ‘the higher ups’

This theme collates participants' experiences with senior management, and explains how those interactions, and what was mandated to them as ward‐based staff, did not always contribute to feeling psychologically safe.

Across interviews, there was a clear sense of polarisation between participants, who were ward‐based staff, and ‘the higher ups’ [Patsy], that is, senior management within participants' respective organisations. Senior management was generally described as absent, not open to communication or interested in hearing constructive feedback. Participants reported working within a culture of blame, feeling afraid to raise concerns and at risk of being penalised for minor mistakes. They suggested that this culture was a barrier to psychological safety.

Psychological safety would be …the people in charge kind of welcoming and wanting to hear feedback, but including critical feedback, and not being defensive about it, but just genuinely wanting to hear it and change.—Hannah.

Some participants described that they felt that raising concerns or asking for changes could put their jobs at risk. This fear was exacerbated in healthcare support workers, who perceived themselves as ‘disposable’ [Patsy] by management. For example, one participant described a situation during the pandemic when hospital management refused the ward COVID‐19 testing kits. The participant wanted to raise this with management but believed that ‘speaking up’ in this way would risk her job security. However, because she was already planning to leave the job role, she felt able to be assertive and demand the tests, to ensure that staff and service users can be protected if necessary.

But that, that's because I didn't need the job that I was able to be like “I don't care if you fire me”—Patsy.

Across interviews, participants expressed that ‘management’ appeared to have no practical insight into the day‐to‐day life on the wards. They also felt management lacked insight into the traumatic nature of some of the incidents that take place on wards, and how this might affect staff's physical and psychological safety.

… under constant scrutiny without actual support of, “How are you coping with this?” It was “Why haven't you done this properly?”—Sarah.

People making decisions without actually working in the wards to know how they're really run. Yes, you can see reports, yes, you can see, you know, this is from admission to bed to medication, the day in, day outs. You don't see how it actually translates, and you don't see why there's such a high turnover of staff, why are there so many complaints, why so many incidents?—Patsy.

[After serious incident], ‘[Management] coming in and saying “You all need to get it together” wasn't the right thing to do’—Sarah.

While participants expressed that they understood that senior ‘management’ of hospitals had to respond to patient safety concerns or incidents, they said that in practice seniors would ‘go on a hunt to find some examples of poor practice in the patient's care’ [Hannah]. They perceived that this approach led to individuals being blamed for their actions, rather than the production of a holistic assessment recognising the constraints in which ward‐based staff operate. It is notable that participants did not distinguish between different levels of management.

Participants reported that they believed that if they were more valued and appreciated by managerial staff, this would facilitate a higher level of psychological safety, even if ward conditions remained the same. Participants acknowledged that incidents are inevitable in inpatient mental health settings due to the nature of the work; but said their psychological safety would be improved if they worked in an ‘environment where you feel safe and supported to raise concerns, and for there to be an environment of sort of, learning from incidents without apportioning blame’ (Hannah). Hence this is one theme that aligns with the traditional definition of psychological safety.

If you make your staff feel valued and appreciated and supported, then actually, although sometimes the situations don't change… that risk level is still there, but we feel more able to handle it […] You were going home after a horrendous shift potentially doing CPR or cutting off 30 ligatures in one night, and just going home and sitting to deal with that on your own, and that… is the problem.—Marie.

Participants also discussed that this lack of care, appreciation and understanding by management reflected the lack of care taken to ensure the safety of physical environments. For example, one participant discussed an incident where a service user managed to access the kitchen and returned with a kettle full of boiling water which she was threatening staff with. The service user was only able to access the kitchen due to a broken door lock ‘that had been reported ages ago’ [Sarah]. Other participants reported of broken alarms, which left their safety compromised in the event of an emergency as they would be unable to alert colleagues to an incident. For participants, these broken fixtures threatened their physical safety and led them to feel undervalued and uncared for by managerial staff.

Thus, an ‘us versus them’ culture, a lack of appreciation and understanding by management, and lack of care to maintain a physically safe environment, were barriers to psychologically safety.

## 4 Theme 5: Support for Staff

The fifth theme collates narratives around staff's ability to access emotional and psychological support for the work they do and the incidents they see or are involved in. There was rarely any support offered to ward‐based staff such as healthcare support workers; only a minority had supervision sessions with ward managers, yet often these did not happen, were ‘rushed’ [Eleanor] and about ‘getting’ frustrations or feelings ‘off my shoulders’ [Daisy] rather than about providing lasting psychological support. Other participants (assistant psychologists, junior doctor) reported access to more structured, regular supervision with seniors, which they found facilitated a sense of psychological safety.

It can be quite traumatic almost working on wards with the things that happen and the things that people see, so […] having spaces to reflect on things and having places to go to seek help if help is needed [to foster psychological safety]—Tatjana.

When support services were offered (e.g., as reflective practice groups or individual counselling after serious trauma), they were often scheduled at unsuitable hours for ward staff, meaning they were unable to attend. Thus, access to programmes or services that could facilitate feelings of psychological safety was not always possible.

People say “Yeah, we really need it, we really want reflective practice” ‐ and then you set up a session and they're like “No‐one's free, sorry.”—Sarah.

Participants welcomed post‐incident debriefs but said they were not always delivered in a helpful way, for example, they might not focus on staff's emotions or feelings, or might not happen at all. Participants identified multiple reasons for this including lack of time, staffing issues, and lack of support from senior management.

It was almost sometimes treated as almost like a fact‐finding session as opposed to actually “OK, so how have you reflected on that? How do you feel about that? Are you, like, are you okay?” I'm not even sure I was asked if I was OK in formal debriefs. […] It just felt like a tick box exercise… it didn't really seem like the people leading them were really that bothered.—Marie.

Establishing access to good psychological support and making debriefs mandatory were suggestions from participants to facilitate psychological safety.

## 5 Discussion

For the inpatient mental healthcare professionals in our study, psychological safety reflected a sense that they were valued in their workplace, protected from physical harm and able to develop meaningful relationships with other staff and service users. Facilitators of psychological safety included feeling physically safe, having an appropriate staffing ratio and skill mix of permanent staff members, being able to form meaningful relationships with patients and having access to support, such as regular supervision. Barriers to psychological safety included reliance on temporary, unexperienced agency workers, punitive management approaches that promote an ‘us versus them’ culture and the physical risk inherent to mental health inpatient contexts. The current findings extend the literature in the following five ways.

First, this study reveals the importance of physical safety in contributing to psychological safety in mental health healthcare. When participants were asked about what makes them feel psychologically safe, participants responded that feeling physically safe is essential to feeling psychologically safe. While participants acknowledged that exposure to risk, violence and aggression is part of their job due to the nature of reasons for admission to acute psychiatric units (see also Bekelepi and Martin 2022; Keers et al. 2018), this study found that exposure to, and experience of, aggression and violence per se affected feelings of psychological safety (even when there was no physical attack). This can be considered a departure point between the concepts of psychological safety in mental healthcare and physical healthcare settings. These findings suggest that Edmondson's traditional definition of psychological safety (Edmondson 1999) does not fit the mental healthcare staff population and needs to be extended to take this into account. Other elements of the traditional definition of psychological safety, such as the notion of trust, remained true. In fact, trust in colleagues and their ability to manage risk situations appropriately was seen as one of the essential components to psychological safety for participants.

Second, previous research has found that staff's feelings of decreased psychological safety are associated with increased risk of patient harm (Grailey et al. 2021). The current study expands this by suggesting that this association may also exist in mental healthcare settings, and is a point of similarity in the concept of psychological safety between mental and physical healthcare settings. Our findings also outline the mechanisms that may underlie this association. For example, exposure to, and experience of, aggression and violence in mental health settings can make staff feel physically and psychologically unsafe, which may lead them to respond differently to patients' behaviour (e.g., physical restraint rather than verbal de‐escalation). This in turn can negatively impact the quality of care given to patients. In this context, it could be suggested that strong leadership, particularly from experienced and well‐trained mental healthcare staff, such as nurses, can aid psychological safety.

Third, by reporting that the presence of temporary workers can also negatively affect the psychological safety of permanent staff, this study extends previous literature that found the presence of temporary workers on mental inpatient units adds to the workloads of permanent staff (Baker, Canvin, and Berzins 2019) and can be a risk‐factor for aggression and violence (Keers et al. 2018; Weltens et al. 2021). While psychological safety has been linked with good teamwork more generally (e.g., Arad et al. 2022; Han and Roh 2020), this study is the first to report the link between temporary staff and (lack of) psychological safety in any healthcare setting. Based on the current results, it can be argued that using agency staff and the over‐reliance on non‐qualified staff to manage an inherently risky patient population is ill‐informed regarding not only patient safety and workloads, but also staff psychological safety.

Fourth, while skill mix and staffing ratio have been linked previously with patient safety, quality of care and mental healthcare staff's intention to leave (Adams, Ryan, and Wood 2021; Baker, Canvin, and Berzins 2019; Keers et al. 2018), this is the first study to highlight that both also play an important role for contributing to psychological safety in mental health healthcare settings. Thus far, the discussion highlights the importance of experienced and well‐trained mental healthcare staff on acute wards, to support staff feeling psychologically safe. In the NHS context, such leadership would typically be provided by senior mental health nurses, whose role it is to not only clinically manage the ward and service users, but also supervise junior staff.

Fifth, this is the first study to provide evidence suggesting that ward staff relationships with senior management and the wider organisation are crucial for developing a sense of psychological safety in mental health staff. The findings reveal a culture of ‘us’ and ‘them’ between the ward staff and the more senior management, which negatively affected psychological safety. Previous research has identified that leadership style and leader–employee relationships are crucial in developing staff's sense of psychological safety in other healthcare settings (Appelbaum et al. 2016; Arnetz et al. 2019; Hirak et al. 2012; Remtulla et al. 2021), and our findings extend this by providing the first evidence that similar relationships appear to be present in acute mental healthcare inpatient settings.

### 5.1 Implications for Practice

The study findings show that staff on mental health wards feel neither physically nor psychologically safe, and this stops them from delivering excellent patient care. It is thus paramount that organisations listen to their staff and invest in ways to make these work environments more physically and psychologically safe.

Across the interviews, participants repeatedly linked a lack of feeling psychologically safe with factors that are known to increase intention to leave, such as lack of just organisational culture, lack of job satisfaction, lack of being able to provide meaningful care, lack of regular supervision, presence of hierarchical structures, and discrepancies between organisational values and the values of staff members (Adams, Ryan, and Wood 2021; Cleary et al. 2012). This is especially salient in the context of the current recruitment and retention crisis in the NHS, where mental health nursing vacancies account for 40% of vacancies (Beech et al. 2019). In addition to the potential negative consequences of working in mental health settings, which include development of trauma, PTSD and burnout (Hilton et al. 2022; Johnson et al. 2018; Needham et al. 2005; Rossi et al. 2012), a lack of psychological safety may exacerbate staffing shortages (Bekelepi and Martin 2022; Johnson et al. 2018; Tane, Fletcher, and Bensa 2022). Further research is needed to identify and evaluate interventions to improve psychological safety, but our findings suggest that providing regular supervision, improving skill‐mix and reducing reliance on temporary staff are all actions which could improve staff's sense of psychological safety.

### 5.2 Limitations

To the best of our knowledge, this study represents the first investigation of staff perceptions of psychological safety in mental health settings. Our sample included both qualified nurses, as well as allied colleagues, such as support workers and assistant psychologists, who play important roles within acute mental health wards. Insights from our findings can be developed in future, larger studies. The strengths of the research are its novel contributions to the literature and its methodological rigour (e.g., multiple researchers familiarising themselves with the data, shared analysis and open discussions regarding the theme development). Limitations of the study included an over‐representation of psychology graduates and assistant psychologists in the participant sample, and an under‐representation of medical doctors and nurses. This is important because previous research has found that mental health nurses, especially, tend to minimise the negative psychological effects of their work environment as a coping strategy (Bekelepi and Martin 2022). The relationships between minimising negative psychological styles and psychological safety are unknown. Further, participants were all‐female identifying, despite extensive efforts to recruit males. This could mean that the emphasis on physical safety in the results may be due to gender (e.g., women feeling more vulnerable than males, or being less physically strong), and therefore the results may not reflect men's experiences.

Staff wellbeing on mental health wards can be a sensitive topic, so to encourage potential participants, we did not ask them to provide personal information other than by completing a consent form and did not capture data regarding where participants worked, physical safety training they had undertaken, or their length of time in practice. These could be interesting lines of inquiry in future studies.

Finally, participants were not given a chance to check their transcripts, or contribute to the analysis. The researchers acknowledge that this is a limitation of the current study, and does not align with the COREQ standards. However, due to the participants being very busy healthcare professionals, it did not seem appropriate to ask them to contribute further to the research.

## 6 Conclusion

This current research shows the important role that physical safety plays in contributing to psychological safety in mental health settings. This means that the traditional definition of psychological safety requires extension for this context, to ensure that it is true to the lived experiences of staff. The research also identified that currently, working in inpatient mental health is not psychologically safe due to organisational factors. Future work should focus on (1) further understanding healthcare staff's conceptualization of psychological safety in inpatient mental health settings; (2) developing an extension of the (Edmondson 1999) measure of psychological safety for inpatient mental health settings, to take the physical safety element into account; and (3) investigating ways to increase psychological safety in inpatient mental health staff via interventions (which should also include measures of intention to leave).

## Author Contributions

The idea for this study was developed by K.S.V., J.J. and J.B. The principal investigator for this study was K.S.V. K.S.V. conducted all interviews. M.M.‐E. transcribed all interviews. K.S.V. led the analysis—supported by M.M.‐E. and K.S.V. wrote the first draft of the analysis and the paper, which J.J., J.B. and S.K. subsequently contributed to. All authors have had the opportunity to read and contribute to the manuscript prior to submission.

## Ethics Statement

Ethical approval for this study was granted by the University of Leeds (Reference: PSYC‐604, approval date 21/10/20220).

## Consent

Participants gave full informed consent before participation. Participants consented that they understood that the experiences shared in the interviews will contribute to the paper, and that direct quotes will be used—but that material would be made as unidentifiable as possible.

## Conflicts of Interest

The authors declare no conflicts of interest.

## Relevance Statement

The concept of psychological safety has gained momentum, with research showing its importance in generating successful outcomes and preventing patient safety incidents/errors. However, it has not been researched in inpatient mental healthcare staff. Participants in this qualitative study conceptualised psychological safety as a feeling of being safe from physical harm, developing meaningful relationships and feeling valued at work. There are two main implications: first, participants did not perceive working in inpatient mental health as psychologically safe, and second, current definitions of psychological safety do not capture psychological safety in the inpatient mental healthcare setting saliently. More research is urgently needed.

## Data Availability

The authors have nothing to report.

## Appendix 1

### Interview Guide for Staff

Can you tell me a little bit about yourself, please?**—**Ascertain what HCP they are, then ask how long they have been a HCP, what setting, etc? Where have you worked before/how long in the present role?

#### Psychological Safety—General

What do you think psychological safety is?—other staff, patients, risk.

#### Personal Experience

Can you tell me about an incident, or incidents, that made you feel psychologically unsafe?—Role of… People? Patients? Yourself? Feelings? Context? Other staff?

Can you tell me about a time that made you feel psychologically safe?—People? Patients? Yourself? Feelings? Context? Other staff?

#### Psychological Safety and Inpatient Psychiatric Wards

What makes a ward psychologically safe in your view, for patients?… or unsafe?—Environment, Other Staff, Patients.

What is the biggest thing that stops wards being psychologically safe for staff?—Environment, Other patients, Staff.

Have you ever done anything while working on the ward, to make yourself feel psychologically safer?

What is the biggest thing that stops wards being psychologically safe for staff?—Environment, Other Staff, Patients.

Are there any things that you think need changing to make wards more psychologically safe?

What things do you think staff should do to encourage psychological safety on wards?

Anything else?
